# Effectiveness of the Combined Approach for Assessing Social Gradients in Stroke Risk Among Married Women in Japan

**DOI:** 10.2188/jea.JE20110147

**Published:** 2012-07-05

**Authors:** Kaori Honjo, Hiroyasu Iso, Masahiko Iwata, Noriko Cable, Manami Inoue, Norie Sawada, Shoichiro Tsugane

**Affiliations:** 1 Global Collaboration Center, Osaka University, Suita, Osaka, Japan; 2 Public Health, Department of Social and Environmental Health, Osaka University Graduate School of Medicine, Suita, Osaka, Japan; 3 Department of Epidemiology and Public Health, University College London, London, United Kingdom; 4 Epidemiology and Prevention Division, Research Center for Cancer Prevention and Screening, National Cancer Center, Tokyo, Japan

**Keywords:** social gradient, measure, stroke, women, Japan

## Abstract

**Background:**

Analysis of the effects of social gradients on women’s health requires a suitable means of assessing social standing.

**Methods:**

We compared social gradients in stroke risk among 9317 married Japanese women from the Japan Public Health Center-based Prospective Study over a 16-year period. Social gradient was estimated by 3 methods of indicating social position: education level derived by using the individual approach (woman’s own educational level), the conventional approach (using her partner’s educational level), and the combined approach (combining the woman’s and her partner’s educational levels).

**Results:**

As compared with the lowest educational group, stroke risk was similar among women in the highest educational group using the individual approach and lower, but not significantly so, with the conventional approach. With the combined approach, however, the age- and area-adjusted hazard ratio (HR) was significantly lower among the highest education group as compared with the lowest group (HR = 0.52, 95% CI: 0.36, 0.76), and the relative index of inequality was significant (RII = 0.48, 95% CI: 0.32, 0.72). Using the combined approach, the results were similar irrespective of employment status. In the combined highest educational group, stroke risk among unemployed women was significantly reduced by 54%, while stroke risk for employed women was significantly reduced by 46%, as compared with the lowest educational group, with RIIs of 0.42 (95% CI: 0.21, 0.85) and 0.49 (0.30, 0.80), respectively.

**Conclusions:**

The results suggest that a combined approach better reflects social standing among married women in Japan.

## INTRODUCTION

Social gradients in health have been well documented by using various socioeconomic indicators such as educational level, income, occupational position, and area deprivation.^1^^,^^2^ However, assessment of social gradients in health among women, especially partnered women, has been a considerable challenge.^3^^–^^13^

The validity of using the social position of spouses or partners as a measure of a woman’s social standing (the conventional approach) has been criticized in light of the increasing number of women entering the labor force.^4^^,^^8^^,^^10^^,^^14^ However, use of a woman’s own social position appears to underestimate social inequalities in health.^15^^,^^16^ Other research has assessed the usefulness of the dominant, or gender-neutral, approach (assessing the most dominant social position in the household) and the combined approach (combining the social positions of all working age adults in the household)^8^ for measuring social gradients in women’s health.^4^^,^^8^^,^^9^^,^^17^

Krieger et al^8^ compared the individual, dominant, and combined approaches and found that social gradients in various health outcomes were greatest using the dominant approach and smallest using the individual approach.

Increasingly large numbers of women in Japan have enrolled in university-level education since 1960.^14^ However, women’s participation in the labor force, by age group, is represented by an M-shape curve, which reflects their tendency to have a career break during their 20s and then return to the labor force, as part-time employees, in their 40s.^14^^,^^18^ In addition, a married woman’s income is unlikely to be similar to her husband’s.^14^^,^^18^

A previous study found that women with the highest and lowest educational levels had higher incidences of total stroke in Japan as compared with those in the middle educational level.^19^ Another study found no significant association between women’s own indicators of social position, such as educational level and occupation, and stroke incidence.^20^ However, information on the relationship between social standing and health among married women is limited.

Krieger et al^8^ concluded that the combined approach was of little use for measuring social gradients in women’s health. However, their findings suggested a need for care in categorizing the social position of married women. Cultural differences might be expected to influence socioeconomic inequalities in women’s health,^21^ and a comparison of the use of these different approaches in non-Western countries could shed new light on the social gradients in women’s health in these countries.

With educational levels as an indicator of social position, we compared social gradients in stroke risk among married women in Japan, using the individual approach (married woman’s own educational level), conventional approach (educational level of her partner), and combined approach (combining educational levels of the woman and her partner). We also investigated the role of women’s employment status in the relationship between educational level and stroke risk.

## METHODS

### Study population

Participants in the Japan Public Health Center-based Prospective Study (JPHC) have been drawn from 4 public health center (PHC) areas since 1990. The recruited participants were 54 498 individuals aged 40 to 59 years, all of whom were registered in 14 administrative districts within those PHC areas. Of the 54 380 eligible participants, 37 851 (70%) completed and returned the self-administered questionnaires at baseline in 1990 and at follow-up in 2000. The sampling design and procedures for the JPHC study have been described in detail elsewhere.^22^ The JPHC study was approved by the institutional review board of the National Cancer Center, Tokyo, Japan.

Women who reported a medical history of cancer, stroke, or myocardial infarction at baseline (*n* = 315) were excluded from the study. We limited inclusion to (1) pairs with an identical surname and address, (2) women with a married partner, and, to eliminate the possibility that an identified pair was a parent and child, (3) pairs with an age difference less than 16 years between the woman and her male partner. A total of 13 292 married couples were identified. Furthermore, to reduce misclassification bias, only women who had remained married for more than 10 years since the baseline survey (*n* = 10 204) were included. Of these women, those with no valid information on their or their partner’s educational level were also excluded (*n* = 572), leaving a total of 9317 married women as our study population.

### Derivation of educational levels using the individual, conventional, and combined approaches

Information on the highest achieved educational level of the married women and their partners was obtained via the baseline questionnaire administered in 1990. Responses were categorized into 3 groups: (1) junior high school (compulsory full-time education in Japan), (2) high school, and (3) college or higher.

Women’s own educational levels were used for the individual approach, while their male partners’ educational levels were used for the conventional approach. For the combined approach, the educational levels of the women and their partners were first categorized as either compulsory education alone or higher education (high school, college, or higher). Four groups were then created: group I (lowest, both partners with compulsory education only), group II (woman with higher education, partner with compulsory education), group III (woman with compulsory education, partner with higher education), and group IV (highest, both partners with higher education).

### Outcome variable

The outcome variable and endpoint for our study was confirmed incidence of total stroke. All study participants were followed for 16 years, from 1990 (baseline) through 2005.

Stroke was defined by using the criteria of the National Survey of Stroke, namely, a neurologic deficit of sudden or rapid onset persisting 24 hours or longer, or until death.^23^ Definitive diagnosis of each stroke subtype, ie, subarachnoid hemorrhage, intraparenchymal hemorrhage, and ischemic stroke (thrombotic or embolic stroke), was established based on clinical findings from computed tomography (CT), magnetic resonance imaging (MRI), or autopsy. A total of 30 hospitals were registered under the 4 PHC areas in the JPHC study. These hospitals were equipped with at least 1 imaging facility (CT or MRI) in a designated clinical cardiology department. The methods used to confirm stroke cases have been described in detail elsewhere.^24^

Residential status and survival were confirmed annually through the residential registry of each PHC area. A total of 645 respondents who moved out of their original area of residence (7%) and 47 married women who were lost to follow-up (0.4%) were treated as censored.

### Covariates

Information on employment status was collected in the baseline survey, and married women were categorized as either employed or unemployed. Information on age, self-perceived psychological status, smoking, physical activity, alcohol use, and existing medical conditions was collected through responses to the baseline questionnaire. Body mass index (BMI) was calculated by using self-reported height and weight at baseline.

Married women were asked to rate their perceived psychological status as low, moderate, or high. Smoking status was recorded as currently smoking, quit smoking, or never smoked. Frequency of leisure time physical activity was recorded as almost none, 1 to 3 times per month, or at least once a week. Alcohol use was categorized as almost none, 1 to 3 times a month, less than 150 g ethanol per week, 151 g to 299 g ethanol per week, and 300 g or more ethanol per week.

Regarding existing health conditions among married women, we identified the presence of hypertension, diabetes, and hypercholesterolemia by examining responses to the relevant questions regarding medical history and/or use of medications to control those conditions. Information on menopausal status was also obtained by examining responses to relevant questions in the baseline questionnaire.

### Statistical analyses

Rates of incident total stroke were calculated during the 16-year follow-up period from 1990 to the endpoint in 2005. Age-adjusted mean values or proportions of cardiovascular risk factors at baseline were calculated based on educational levels derived from the 3 approaches by using multivariate regression for continuous measures and logistic regression for dichotomous measures.

Hazard ratios (HRs) with 95% CIs were calculated using Cox proportional hazards regression after adjusting for age and area, which were regarded as potential confounding variables (Model 1). Further adjustments were made for known conventional cardiovascular risk factors such as smoking, alcohol use, perceived psychological stress, physical activity, BMI, history of hypercholesterolemia, hypertension, diabetes, and menopause, which were considered mediators in this study (Model 2).

A relative index of inequality (RII) was calculated for each model to obtain a quantitative estimate of the overall magnitude of inequalities in the stroke risk of married women. The RII is a regression-based summary measure used in research on social inequalities.^25^ Dummy variables for hierarchical categories of social position were given to each individual (low = 0.1, medium = 0.5, high = 0.9 for both the conventional and individual approaches; Q1 = 0.05, Q2 = 0.35, Q3 = 0.65, Q4 = 0.95 for the combined approach). The RII encompasses a range from 0 (lowest) to 1 (highest), weighted by the number of subjects in each category, and represents the ratio of the stroke risk for those at the bottom of the social hierarchy versus those at the top of the hierarchy.^26^^,^^27^ Further analysis was conducted after stratification according to women’s employment status. Married women without information on employment status were excluded from this stratified analysis (*n* = 71). These analyses were applied to all 3 approaches. All analyses were conducted using SAS version 9.2.

## RESULTS

Among our study sample of married Japanese women (*n* = 9317), there were 179 cases of newly diagnosed stroke during 144 655 person-years of follow-up (mean follow-up, 15.5 years). Table 1 shows the distribution of educational levels of male partners by the educational level of the married women. These data suggest that men and women were likely to find partners with the same educational level, though the proportion of such marriages declined with increasing educational level.

**Table 1.** Numbers of married women (*n* = 9317) at different educational levels according to their partner’s educational level

	Partner’s educational level		

	Junior high school (*n* = 4335)	High school (*n* = 3792)	College or higher (*n* = 1190)
Women’s educational level			
Junior high school			
(*n* = 4429, %)	3156 (71)	1152 (26)	121 (3)
High school			
(*n* = 3763, %)	1014 (27)	2208 (59)	541 (14)
College or higher			
(*n* = 1125, %)	165 (15)	432 (38)	528 (47)

Table 2 shows the demographic characteristics of married women according to educational level, as derived from the 3 approaches. Regardless of the approach used, women with the highest educational level were more likely to be young, stressed, and physically active and were less likely to smoke, be obese, have a history of hypertension, or be menopausal than women in the lowest educational group. The proportions of employed women were similar in all educational groups, though slightly more women who were partnered with a college-educated man were not employed.

**Table 2.** Age-adjusted baseline characteristics according to educational level derived from individual, conventional and combined approaches among a cohort of married women (*n* = 9317)

	Individual approach			Conventional approach			Combined approach			
		
	JHS	HS	College	JHS	HS	College	Group I (lowest) JHS:JHS	Group II HS/College:JHS	Group III JHS: HS/college	Group IV (highest) HS/college: HS/college
No. at risk	4429	3763	1125	4335	3792	1190	3156	1179	1273	3709
Mean age (year)	49.2	47.4	46.3	49.0	47.5	46.9	49.5	47.9	48.8	46.9

Employed (%)	75.5	75.9	75.8	76.9	76.7	68.1	76.6	77.4	72.9	75.3
Perceived high psychological stress (%)	18.3	23.4	33.6	19.6	23.0	28.5	18.4	23.1	18.2	26.6
Current smoker (%)	4.1	2.9	3.1	3.9	3.2	3.0	3.8	4.0	4.7	2.7
Ethanol consumption >300 g/week (%)	0.7	0.7	0.1	0.7	0.5	0.4	0.7	0.8	0.7	0.4
Physical activity more than once a week (%)	13.3	17.5	21.3	13.3	17.6	20.6	12.4	15.6	15.6	19.3
Mean BMI (kg/m^2^)	23.7	23.2	23.1	23.7	23.3	22.9	23.8	23.4	23.6	23.1
BMI ≥27 (%)	14.0	9.6	9.0	13.6	10.7	7.6	14.5	11.2	12.7	8.9
Hypercholesterolemia (%)	1.5	1.5	1.0	1.5	0.6	0.9	1.7	1.1	1.1	1.5
Medical history of hypertension (%)	13.1	10.3	9.4	13.2	10.3	9.9	13.6	12.2	12.1	9.4
Medical history of diabetes mellitus (%)	2.2	1.3	1.9	1.6	1.6	1.3	1.8	1.2	2.0	1.3
Menopause (%)	40.2	38.7	35.2	40.5	37.9	36.6	40.6	40.6	39.2	37.1

JHS = junior high school; HS = high school.

BMI = Body Mass Index (Kg/m^2^).

Table 3 presents the adjusted HRs and 95% CIs for stroke risk among married women according to educational levels derived using the individual and conventional approaches. Analysis using the woman’s educational level (individual approach) yielded a U-shaped association. Regarding the compulsory education group, age- and area-adjusted HRs for stroke risk among women in the high school education group and the college/higher education group were 0.56 (95% CI: 0.39, 0.80) and 0.99 (0.61, 1.58), respectively. The calculated RII was not significant for the individual approach.

**Table 3.** Hazard ratios (HRs) with 95% CIs for stroke risk in married women (*n* = 9317) according to educational levels derived from individual and conventional approaches

		Individual approach				Conventional approach			
	
		Junior high school	High school	College	RII^a^	Junior high school	High school	College	RII^a^
ALL	Person years	69 138	58 362	17 155	144 655	67 712	58 865	8078	144 655
	No. of cases	114	44	21	179	115	46	18	179
Model 1	HR	1.00	0.56 (0.39, 0.80)	0.99 (0.61, 1.58)	0.62 (0.34, 1.12)	1.00	0.54 (0.39, 0.77)	0.74 (0.45, 1.23)	0.45 (0.25, 0.83)
Model 2	HR	1.00	0.61 (0.43, 0.88)	1.09 (0.67, 1.76)	0.73 (0.40, 1.32)	1.00	0.59 (0.41, 0.84)	0.82 (0.49, 1.36)	0.53 (0.29, 0.96)

Unemployed^b^	Person years	17 576	13 390	3761	34 728	16 241	13 105	5381	34 728
	No. of cases	40	12	10	62	40	12	10	62
Model 1	HR	1.00	0.47 (0.25, 0.88)	0.79 (0.33, 1.80)	0.40 (0.14, 1.19)	1.00	0.43 (0.23, 0.83)	0.94 (0.46, 1.90)	0.57 (0.22, 1.48)
Model 2	HR	1.00	0.50 (0.27, 0.96)	0.81 (0.34, 1.96)	0.46 (0.16, 1.36)	1.00	0.47 (0.24, 0.91)	1.03 (0.50, 2.13)	0.66 (0.25, 1.74)

Employed	Person years	51 018	44 549	13 233	108 801	50 894	45 338	12 568	108 801
	No. of cases	75	34	8	117	75	34	8	117
Model 1	HR	1.00	0.60 (0.39, 0.93)	1.11 (0.63, 1.96)	0.74 (0.36, 1.52)	1.00	0.59 (0.36, 0.89)	0.55 (0.26, 1.14)	0.36 (0.16, 0.78)
Model 2	HR	1.00	0.65 (0.42, 1.02)	1.22 (0.68, 2.18)	0.86 (0.42, 1.79)	1.00	0.64 (0.42, 0.97)	0.60 (0.29, 1.27)	0.42 (0.19, 0.93)

Model 1: age- and area-adjusted.

Model 2: Model 1 + conventional cardiovascular risk factors (smoking status, alcohol use, perceived psychological stress, physical activity, Body Mass Index, history of hypercholesterolemia, hypertension, diabetes, and menopause).

^a^RII = Relative Index of Inequality; ^b^Unemployed = economically inactive women.

HR = Hazard Ratio.

Using the conventional approach, the age- and area-adjusted HR for stroke risk among women in the high school education group was significantly reduced by 46%, relative to those in the lowest educational group (HR = 0.54; 95% CI: 0.39, 0.77), and remained significantly lower even after controlling for hypothesized mediating factors. The age- and area-adjusted stroke risk was also lower among women in the college/higher educational level group, but not significantly so (HR = 0.74; 0.45, 1.23). The age- and area-adjusted RII calculated by using the conventional approach was steeper than that obtained using the individual approach (RII = 0.45; 95% CI: 0.25, 0.83).

We further tested the effect of employment status on the associations between educational levels and stroke risk among married women. The associations between derived educational level and stroke risk differed according to employment status. Using the individual approach, employment status had no effect on the association between educational level and stroke risk: a U-shaped association was identified for both employed and unemployed women. However, employment status did influence the association between stroke risk and partner’s educational level (conventional approach): age- and area-adjusted stroke risk was higher in unemployed women with a college level or higher education, as compared with unemployed women with a high school education. There was a U-shaped association between stroke risk and partner’s educational level among unemployed women. A different pattern was observed in employed women, ie, stroke risk decreased among employed women as their partner’s educational level increased. The age- and area-adjusted RII was significant (RII = 0.36; 95% CI: 0.16, 0.78).

Using the combined approach, married women’s stroke risk was lower in all groups, as compared with the lowest educational group (Table 4). The age- and area-adjusted HRs for stroke risk were 0.71 (95% CI: 0.44, 1.14) for Group II, 0.58 (0.36, 0.95) for Group III, and 0.52 (0.36, 0.76) for Group IV (both with more than compulsory education) as compared with Group I (both with compulsory education only). Adjusting for conventional cardiovascular risk factors slightly attenuated the HRs for those educational groups, but the pattern of association was unchanged, and the effects observed in Groups III and IV remained significant. The age- and area-adjusted RII obtained using this approach was similar to that obtained using the conventional approach and was statistically significant (RII = 0.48, 95% CI: 0.32, 0.72).

**Table 4.** Hazard ratios (HRs) with 95% CIs for stroke risk in married women (*n* = 9317) according to combined (woman:partner) educational level

		Combined approach				

		Group I (lowest) JHS:JHS	Group II HS/college:JHS	Group III JHS:HS/College	Group IV (highest) HS/college:HS/college	RII^a^
ALL	Person years	49 299	18 413	19 839	57 103	144 655
	No. of cases	94	21	20	44	179
Model 1	HR	1.00	0.71 (0.44, 1.14)	0.58 (0.36, 0.95)	0.52 (0.36, 0.76)	0.48 (0.32, 0.72)
Model 2	HR	1.00	0.74 (0.46, 1.20)	0.61 (0.37, 0.99)	0.59 (0.40, 0.86)	0.54 (0.36, 0.81)

Unemployed^b^	Person years	12 106	4135	5470	13 017	34 728
	No. of cases	35	5	8	14	62
Model 1	HR	1.00	0.49 (0.19, 1.26)	0.59 (0.27, 1.27)	0.46 (0.24, 0.87)	0.42 (0.21, 0.85)
Model 2	HR	1.00	0.49 (0.19, 1.26)	0.59 (0.27, 1.30)	0.51 (0.26, 0.99)	0.47 (0.23, 0.96)

Employed	Person years	36 761	14 133	14 257	43 649	108 801
	No. of cases	59	16	12	30	117
Model 1	HR	1.00	0.81 (0.46, 1.42)	0.56 (0.30, 1.04)	0.54 (0.34, 0.86)	0.49 (0.30, 0.80)
Model 2	HR	1.00	0.84 (0.48, 1.49)	0.58 (0.31, 1.09)	0.61 (0.38, 0.98)	0.55 (0.33, 0.92)

Model 1: age- and area-adjusted.

Model 2: Model 1 + conventional cardiovascular risk factors (smoking status, alcohol use, perceived psychological stress, physical activity, Body Mass Index, history of hypercholesterolemia, hypertension, diabetes, and menopause).

^a^RII = Relative Index of Inequality; ^b^Unemployed = economically inactive women.

HR = Hazard Ratio; JHS = junior high school; HS = high school.

Stratification by married women’s employment status yielded information on the effect of their employment on the relationship between combined educational levels and their stroke risk. Among highly educated women whose partner was only educated to compulsory level (Group II), stroke risk was reduced by 50% if they were unemployed, but by only 19% if they were employed. When highly educated women were partnered with a man from the same educational background (Group IV), stroke risk was lower in unemployed women (HR = 0.46, 95% CI: 0.24, 0.87) than in employed women (0.54, 0.34, 0.86). The effect of women’s employment status on their stroke risk was marginal if they were only educated to compulsory level and were partnered with a highly educated man.

## DISCUSSION

Analysis using educational levels derived from the individual, conventional, and combined approaches gave different views of the association between educational level and stroke risk among married women in Japan. Education level, as ascertained by using the women’s own educational levels (individual approach) or their partner’s educational levels (conventional approach), was nonlinearly associated with stroke risk in married women, while educational level derived by combining the educational levels of women and their partners (combined approach) identified a significantly lower stroke risk among women in all educational groups as compared with the lowest educational group. The RII obtained via the combined approach was greater than that based on individual educational level and was significantly greater than that based on conventional educational level. These results demonstrate the usefulness of educational level as a measure of social position, when educational level is assessed with the combined approach.

Studies in Europe^6^^,^^15^^,^^16^ and the United States^8^^,^^28^ showed that social gradients in women’s health were likely to be underestimated when women’s social position was based on their own standing. Our findings confirmed that the social gradient in married women’s stroke risk was smallest when using the individual approach. The nonlinear, U-shaped association between educational level and stroke risk identified in our study was similar to that found in our previous study^19^ and suggests that socioeconomic indicators for women such as educational level, occupational position, and earned income may not reflect their place in hierarchal society, thus minimizing the apparent effect of social gradients on the health of women.

The age- and area-adjusted RII was significant for the conventional and combined approaches; however, the combined approach produced a linear social gradient in stroke risk, while the conventional approach did not. Although Krieger et al^8^ gave the combined approach little credit because of the consequent reduction in statistical power, the results of the current study strongly suggest the need for careful examination of each combined group before consolidation.

Stratification according to married women’s employment status added another dimension to the association between educational level and stroke risk. Analysis using the individual and conventional approaches yielded a U-shaped relationship in unemployed women, while the conventional and combined approaches showed a linear relationship for employed women. This suggests that socioeconomic conditions in each household are likely to be influenced by the partner’s educational level, over and above the women’s own level. The identification of an effect of social gradient on stroke risk using the combined approach, regardless of employment status, confirms the effectiveness of this approach in reflecting the psychosocial context of stroke risk among married women.

We adjusted for conventional cardiovascular risk factors to determine if those factors could explain the identified social gradient in stroke risk. However, adjustment for cardiovascular risk factors did not materially attenuate the association between social position and risk of stroke, which suggests that these risk factors could not explain the social gradient. Further studies are needed to explore the mechanisms underlying the social gradient in stroke risk.

There were some limitations to the current study. Our findings may be limited to married women living in medium and small cities in Japan. Some variables were measured only once at baseline, and we relied on self-report, which possibly led to misclassification. The findings may also be limited by the criteria we used to establish marital status and identify total stroke cases. To minimize the risk of misclassification, we excluded women who were no longer married 10 years after the baseline survey. In addition, information on the educational levels of partners was obtained from valid responses by the corresponding male partner. The study protocol might have reduced the numbers in the study population, though any potential bias in the results is likely to be small.

In summary, a combined measure of educational level, especially when stratified by women’s employment status, could provide a detailed profile of the association between social gradient and stroke risk among married women in Japan. Individual or conventional measures of social position are likely to underestimate the magnitude of social gradients in women’s health, thus hampering the development and implementation of relevant policies specific to women.

## References

[1] Siegrist J, Marmot M. Introduction— Social inequalities in health: basic facts. In: Siegrist J, Marmot M, editors. Social inequalities in health. Oxford: Oxford university press; 2006. p. 1–25.

[2] Lynch J, Kaplan G. Socioeconomic position. In: Berkman LF, Kawachi I, editors. Social epidemiology. New York: Oxford university press; 2000. p. 13–35.

[3] Acker J Women and social stratification: a case of intellectural sexism. Am J Sociol. 1973;78(4):936–45 10.1086/225411

[4] Goldthorpe JH Women and class analysis: In defense of the conventional view. Sociology. 1983;17(4):465–88 10.1177/0038038583017004001

[5] Krieger N, Williams DR, Moss NE Measuring social class in US public health research: concepts, methodologies, and guidelines. Annu Rev Public Health. 1997;18:341–78 10.1146/annurev.publhealth.18.1.341 9143723

[6] Moser KA, Pugh HS, Goldblatt PO Inequalities in women’s health: looking at mortality differentials using an alternative approach. Br Med J (Clin Res Ed). 1988;296(6631):1221–4 10.1136/bmj.296.6631.1221 3133022 PMC2545703

[7] Macintyre S, McKay L, Der G, Hiscock R Socio-economic position and health: what you observe depends on how you measure it. J Public Health Med. 2003;25(4):288–94 10.1093/pubmed/fdg089 14747587

[8] Krieger N, Chen JT, Selby JV Comparing individual-based and household-based measures of social class to assess class inequalities in women’s health: a methodological study of 684 US women. J Epidemiol Community Health. 1999;53(10):612–23 10.1136/jech.53.10.612 10616673 PMC1756781

[9] Shirahase S Women and class structure in contemporary Japan. Br J Sociol. 2001;52(3):391–408 10.1080/00071310120071115 11578002

[10] Galobardes B, Shaw M, Lawlor DA, Davey Smith G, Lynch JW. Measures of Socioecnomic Position. In: Oakes JM, Kafufman JS, editors. Method in social epidemiology. San Francisco: Jossey-Bass; 2006. p. 47–85.

[11] Berkman LF, Macintyre S The measurement of social class in health studies: old measures and new formulations. IARC Sci Publ. 1997;(138):51–64 9353663

[12] Chandola T, Bartley M, Wiggins R, Schofield P Social inequalities in health by individual and household measures of social position in a cohort of healthy people. J Epidemiol Community Health. 2003;57(1):56–62 10.1136/jech.57.1.56 12490650 PMC1732272

[13] Bartley M, Martikainen P, Shipley M, Marmot M Gender differences in the relationship of partner’s social class to behavioural risk factors and social support in the Whitehall II study. Soc Sci Med. 2004;59(9):1925–36 10.1016/j.socscimed.2004.03.002 15312926

[14] Shirahase S. Univisible inequalities in an aging society with fewer children. Tokyo: Tokyo daigaku syuppankai; 2005.

[15] Chandola T Social inequality in coronary heart disease: a comparison of occupational classifications. Soc Sci Med. 1998;47(4):525–33 10.1016/S0277-9536(98)00141-5 9680236

[16] Sacker A, Firth D, Fitzpatrick R, Lynch K, Bartley M Comparing health inequality in men and women: prospective study of mortality 1986–96. BMJ. 2000;320(7245):1303–7 10.1136/bmj.320.7245.1303 10807620 PMC27373

[17] Naoi M. Social class. In: Okamoto H, Naoi M, editors. Social sructure of Japanese society 4—Women and social class. Tokyo: Tokyo daigaku syuppankai; 1990. p. 147–64.

[18] Tachibanaki T. Inequalities among women. Tokyo: Toyokeizaishinpousha; 2008.

[19] Honjo K, Iso H, Inoue M, Tsugane S; the JPHC Study Group Education, social roles, and the risk of cardiovascular disease among middle-aged Japanese women: the JPHC Study Cohort I. Stroke. 2008;39(10):2886–90 10.1161/STROKEAHA.108.514067 18658036

[20] Honjo K, Tsutsumi A, Kayaba K; Jichi Medical School Cohort Study Group Socioeconomic indicators and cardiovascular disease incidence among Japanese community residents: the Jichi Medical School Cohort Study. Int J Behav Med. 2010;17(1):58–66 10.1007/s12529-009-9051-7 19554455

[21] Martikainen P Women’s employment, marriage, motherhood and mortality: a test of the multiple role and role accumulation hypotheses. Soc Sci Med. 1995;40(2):199–212 10.1016/0277-9536(94)E0065-Z 7899932

[22] Tsugane S, Sobue T Baseline survey of JPHC study—design and participation rate. Japan Public Health Center-based Prospective Study on Cancer and Cardiovascular Diseases. J Epidemiol. 2001;11(6 Suppl):S24–9 10.2188/jea.11.6sup_24 11763136 PMC11858361

[23] Walker AE, Robins M, Weinfeld FD The National Survey of Stroke. Clinical findings. Stroke. 1981;12(2 Pt 2 Suppl 1):I13–44 7222164

[24] Iso H, Baba S, Mannami T, Sasaki S, Okada K, Konishi M, Alcohol consumption and risk of stroke among middle-aged men: the JPHC Study Cohort I. Stroke. 2004;35(5):1124–9 10.1161/01.STR.0000124459.33597.00 15017008

[25] Mackenbach JP, Kunst AE Measuring the magnitude of socio-economic inequalities in health: an overview of available measures illustrated with two examples from Europe. Soc Sci Med. 1997;44(6):757–71 10.1016/S0277-9536(96)00073-1 9080560

[26] Davey-Smith G, Dorling D, Mitchell R, Shaw M Health inequalities in Britain: continuing increases up to the end of the 20th century. J Epidemiol Community Health. 2002;56(6):434–5 10.1136/jech.56.6.434 12011199 PMC1732168

[27] Singh-Manoux A, Dugravot A, Shipley MJ, Ferrie JE, Martikainen P, Goldberg M, The association between self-rated health and mortality in different socioeconomic groups in the GAZEL cohort study. Int J Epidemiol. 2007;36(6):1222–8 10.1093/ije/dym170 18025034 PMC2610258

[28] Krieger N Women and social class: a methodological study comparing individual, household, and census measures as predictors of black/white differences in reproductive history. J Epidemiol Community Health. 1991;45(1):35–42 10.1136/jech.45.1.35 2045742 PMC1060699

